# Screen Time and Attention Subdomains in Children Aged 6 to 10 Years

**DOI:** 10.3390/children9091393

**Published:** 2022-09-15

**Authors:** Magnus Liebherr, Mark Kohler, Julia Brailovskaia, Matthias Brand, Stephanie Antons

**Affiliations:** 1 Department of General Psychology: Cognition & Center for Behavioral Addiction Research, University of Duisburg-Essen, Magnus Liebherr, Forsthausweg 2, 47057 Duisburg, Germany; 2 Erwin L. Hahn Institute for Magnetic Resonance Imaging, 45141 Essen, Germany; 3 School of Psychology, University of Adelaide, Adelaide, SA 5005, Australia; 4 Department of Clinical Psychology and Psychotherapy, Mental Health Research and Treatment Center, Ruhr-Universität Bochum, 44801 Bochum, Germany

**Keywords:** children, digital media, attention, development, cognition

## Abstract

Using digital media has become the most popular leisure activity for children and adolescents. The effects of digital media use on the developing brain and cognitive processes of children are subject to debate. Here, we examine the effect of digital media use on attention subdomains in children aged 6 to 10 years. In total, 77 children participated in the study. Selective and divided attention as well as switching between attentional subdomains were quantified by the SwAD-task. Parents were asked to assess the screen time of their children (smartphone, laptop/PC, game console, tablet, TV). Results show no main or interaction effects of screen time on any of the attention conditions investigated. Based on the present findings as well as previous studies, we suggest a possible non-linear relationship between the amount of screen time and attention function. Furthermore, we emphasize the relevance of considering the socio-economic background of children and a need for longitudinal studies.

## 1. Introduction

More than half a century ago, televisions entered the households of children around the globe, followed by game consoles in the 1970s. Today, more than one-third of children have their own smartphone or tablet device [1,2,3], with younger children being among the strongest users of digital media applications [4]. However, their developmental vulnerability makes it necessary to critically consider the effects of screen time, especially in this age group. In available literature, effects have been heatedly discussed in different areas, such as mental and physical health [5], social skills [6], as well as cognitive functions [7]. Findings show consistent evidence about negative effects mainly depending on both the duration and content of media use [8,9].

In earlier research, effects of using digital media on cognitive abilities been discussed controversial [7,9,10,11]. This topic is further complicated because cognitive abilities comprise a variety of mental processes such as attention, perception, inhibition, and decision making. Therefore, in addition to the duration and content, individual cognitive functions need to be considered specifically to improve our understanding of the effects of digital media use. In the present study, we focus on the effects of screen time on attention: the ability to sort and focus on relevant stimuli.

Previous studies on watching television in early childhood identified a negative relation between watching television and attention skills, especially when watching programs created for adults [10]. With regard to attention problems, some identified a relation with watching television [12,13,14], while others did not [15,16,17,18]. In those who identified significant relationships, the amount of screen time related positively to attention problems varied from >1 h/day [12] to >2 h/day [13] of watching television in young children. In adolescence, the value for which negative effects on attention are reported increases to >3 h/day [19].

In contrast to watching television, which represents a passive way of using (digital) media, computers, tablets, and smartphones allow children to increasingly interact with the devices and applications. Video games are one of the applications most commonly used by children. They have even been reported to generally improve attention skills [20]. More specifically, findings from children/teenagers of different ages show improvements in alerting and orienting [21], selective visual attention [22], as well as sustained attention [23] related to playing action video games. However, evidence from clinical studies shows a positive relationship between pathological gaming behavior and attention problems [24]. Similar findings have been reported in clinical studies on problematic smartphone use [25]. However, in both, there is also evidence showing no relationship between video-gaming and attention problems [26,27].

In children/adolescents, smartphones are most frequently used for communication applications (phone calls, social networking apps, messenger apps) and Internet browsing in addition to listening to music and watching videos [28]. Counterintuitively, findings show a relationship between heavy use of social networking site/Internet browsing and improvements in attention performance in children and adolescents [29]. However, there is, to our best knowledge, no further evidence on the effects of smartphone usage on attention processes in children/adolescents. However, evidence from adult samples show an increased level of distraction/inattention in the mere presence of a smartphone, but longitudinal studies are also missing [30].

Digital devices are constantly evolving, and so are their applications. As a result, demands and possible effects on attention processes are also permanently changing, which calls for a regular update of our understandings in the present field of interest. Especially in children, findings on the effects of using digital media on subcomponents of attention are limited. Here, the present study aims to contribute by providing findings on the relationship between using different digital media applications (television, laptop/computer, tablet, smartphone) and subdomains of attention in primary school-aged children. We focus on the most relevant attention subdomains of everyday life: switching attention, divided attention, and selective attention [31].

## 2. Methods

### Participants

In total, 77 children (age: *M* = 8.04 years, *SD* = 1.35; range: 6–10 years; 35 girls; 1st grade–4th grade) participated in the present study. All were reported by parents to have normal or corrected-to-normal hearing and vision and to have no history of psychological and/or neurological disorders or any acute disease. Both children and parents were informed about the study, with parents providing consent and children assenting for participation. Children were further informed that they could end the study at any time. The study was performed in accordance with the ethical standards laid down in the Declaration of Helsinki and approved by the local ethics committee of the Department of Computer Science and Applied Cognitive Sciences, University Duisburg-Essen.

## 3. Materials

### 3.1. Attention Subdomains

A modified version of the Switching Attentional Demands (SwAD) task was used to measure performance of selective and divided attention as well as switching between both subdomains [32]. The modification refers to the visual stimuli presented. In the present context, we used simple symbols instead of geometric shapes to make the task less complex for administration to children.

**Figure 1:**
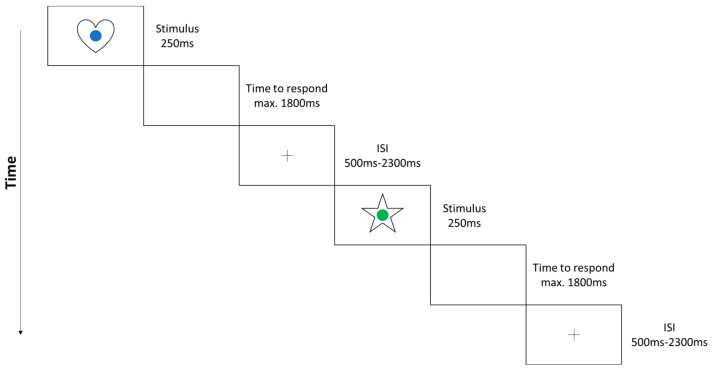
Overview of the SwAD-task procedure (child version) (see also Liebherr et al., 2019).

The task comprises a training session that provides feedback as well as four blocks of selective attention, four blocks of divided attention, and eight blocks of switching attention. Each block includes 26 trials with five to eight target stimuli. Stimuli include colored dots (e.g., blue, red, green) and symbols (e.g., heart, moon, star, flower), each presented for 250 ms. The respective symbol as well as colored dot were presented simultaneously in the middle of the screen (see Figure 1). The two conditions differed solely in their instruction. In selective attention, participants should respond to either a colored dot (e.g., blue) or a shape (e.g., flower) by pressing a button. In divided attention, both colored dots and shapes acted as target stimuli to which participants had to respond by pressing different buttons. In switching attention, four blocks of selective attention and four blocks of divided attention were applied, alternating with no break in between. Interstimulus intervals were randomized between 500 ms and 2300 ms, in which a fixation-cross was presented in the middle of the screen. The maximum time to respond to a single stimulus was set to 1800 ms. Total time for the task was approximately 20 min. Reaction time was used to quantify task performance, while error rate was used to identify outliers [32].

### 3.2. Digital Media Use and Leisure Activity

Digital media use was measured using an online survey. Parents were asked if the children have their own television, smartphone, tablet, game console, or computer as well as how much they use it on average each day. Ratings were made on a scale from none, <30 min, 30 min, 1 h, 2 h, 3 h, to >4 h separately for each media device as would be typical for a regular day. In addition, all children were asked after their testing which games they usually play on the PC/game console and which applications they use on their smartphone. Furthermore, they were asked which non-digital activities are important to them in their leisure time and whether they go to a care facility in the afternoon or not.

## 4. Procedure

Participants were recruited through a local elementary school (May–July 2020). The children were informed about the study and asked if they would like to participate. In the event of acceptance, the parents were informed and provided written consent. Together with the information, the parents received a link to the online survey where data regarding the age, gender, school level, and media usage of the child were obtained. In order to be able to match the data of the online survey with the data of the laboratory study and still guarantee anonymity, codes were assigned in advance. The laboratory part of the study took place in an empty room at the children’s school to keep the effort for the children as low as possible. A usual classroom table and chair were positioned in a standardized position. An external monitor was used to present the tasks. The upper edge of the screen was set at the participant’s eye level. In addition, a keyboard for answering the tasks was placed on a predefined position on the table in front of the participant. All instructions were read to the children to ensure that the tasks were understood. At the end of all tasks, each child received a small present for participating.

## 5. Statistical Analysis

The statistical analyses were computed using SPSS 26.0 for Windows (IBM SPSS Statistics, released 2019). Outliers were excluded prior to mean calculations. For this, error rates of each block that exceeded two standard deviations of a participant’s mean of the respective block were identified. After that, we calculated mean reaction times for selective and divided attention in single demand and switching conditions.

Pearson’s correlations were calculated for associations between media use and attentional subdomains.

## 6. Results

### 6.1. Descriptive Statistics

None of the children attended a care facility in the afternoon. Within their leisure time, 92% met up with friends, 91% played sports, and 78% found it important to spend time with their families. In addition, 66% did handcraft, and 65% read regularly. Listening to music was mentioned by 62% as an activity in their leisure time, and 27% liked to go shopping.

Roughly half of the children owned a game console (57%), a smartphone (43%), and/or a tablet (48%). Significantly less owned a computer/laptop (16%) and/or a TV (19%). However, all children watched TV regularly, with 66.2% of participants watching one hour or less per day. About half of all children did not use a smartphone (50.6%), tablet (49.4%), or game console (40.3%). Most children (76.6%) did not use a computer regularly. Media use of all children participating in the study was reported as restricted by their parents. Mean media usage time in hours per day as well as minimum and maximum values are reported in Table 1.

**Figure 2:**
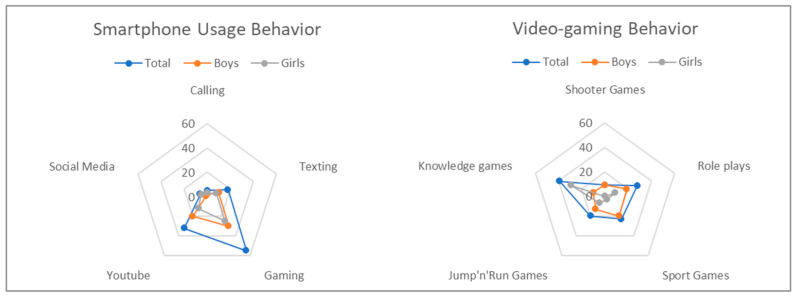
Usage behavior for single applications in gaming and smartphone use for the total sample and divided by gender.

In addition, smartphone usage behavior shows that the children use the device mainly for gaming and watching YouTube videos. Only a small percentage uses it for texting, social media, and calling. In videogaming, knowledge games are the most popular, followed by role-playing games, sports games, and platform games (see also Figure 2).

**Figure 3:**
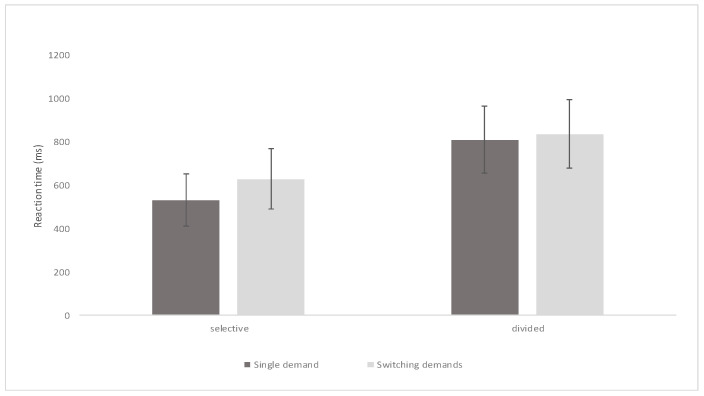
Means and standard deviations for attention subdomains.

Descriptive findings from the SwAD-task on attention subdomains of selective attention, divided attention, and switching attention are presented in Figure 3. Due to dropouts in specific tasks, analyses are based on data from 61 children.

### 6.2. Associations between Media Use and Attentional Subdomains

There were no significant correlations between media use and attentional subdomains as measured with the SwAD-task (see Table 2). Due to low variances in the use of specific media within the present sample (all participants had more or less the same low-usage times), no correlations were calculated between attentional subdomains and the use of TV, smartphone, tablet, game console, and computer.

## 7. Discussion

The use of digital media is becoming increasingly prevalent amongst children, with its use extended to younger and younger ages [1,3]. However, an understanding of the effects of using digital media, such as smartphones, tablets, etc., on the development of cognitive functions is still limited. Therefore, the major focus of the current study was to investigate the effect of digital media use on subdomains of attention. While previous studies show an influence of using digital media on cognitive functions [1,33], we did not identify any effect on attentional subdomains.

**Figure 4:**
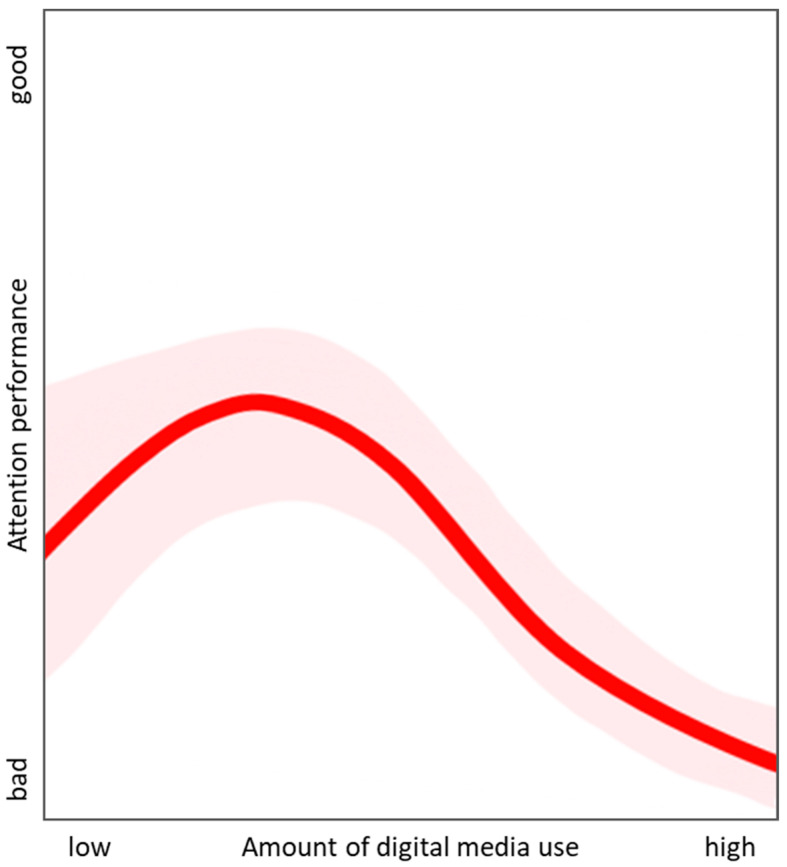
Suggestion of a non-linear relationship between the amount of digital media use and cognitive functions/attention performance. Assuming a variation in the effects depending on the content, as well as intra- and interpersonal factors, such as the family, the social environment, as well as the individual characteristics of the children (shown in light red).

However, the study design and effects shown do provide valuable information to the field. Together with previous findings—that show specific improvements in cognitive functions for certain applications [20,21,34]—we suggest a possible non-linear relationship between the amount of digital media use and cognitive functions that requires investigation (see Figure 4). The proposal is that digital media in a lower- or middle-intensity range, as in the present study, could have no or even positive effects on attention performance depending on the content (i.e., domains are trained by using corresponding digital media applications whose content is closely related to them). However, as soon as the use becomes excessive, or there is a tendency towards problematic or pathological use, the positive effects turn into the opposite.

The most promising evidence supporting our suggestion comes from studies on videogaming. Here, studies describe lower hyperactivity, fewer conduct problems, and fewer internalizing and externalizing problems for low-use gaming [35,36].

Further evidence comes from studies focusing on mental health and psychological well-being in the context of using digital media. Here, Twenge and colleagues advocated an exposure–response curve hypothesis that originates in the context of alcohol and marijuana [37,38]. The authors assume that well-being peaks at light use and progressively lowers as digital media use moves from light to moderate to heavy.

In the present sample, we did not assess the socio-economic background but recruited participants solely in one school, with children from surrounding areas, and did not control for single- vs. two-parent families. The school is located in a suburb with relatively high land prices and exclusively single-family homes. It can therefore be assumed that the group examined consists of children with a relatively high socio-economic background, representing a very homogeneous group. The lack of an explicit consideration of the socio-economic background, however, represents a limitation. Therefore, future studies on media use in children and adolescents should increasingly consider aspects such as maternal education, child poverty, and parents monitoring as potential covariates. Another limitation was pointed out to us by the reviewers. This relates to the screen time assessment. We asked parents for their assessment. However, recent studies show that this does not correspond to the objective usage time [39].

Given the low variance in overall media use in hours per day and the lack of association with attention subdomains, we did not test the individual effects of television, smartphone, tablet, game console, and computer. Future studies should address this limitation by investigation such associations in a broader range of children with differing media usage behavior. Furthermore, future studies should aim to investigate further subdomains of switching attention, such as switching between different modalities, spaces, attributes, and stimulus/response sets. In order to address the limitations above, we suggest the need to undertake longitudinal considerations in future studies to provide a deeper understanding of cause-and-effect relationships as well as more accurately track developmental trajectories.

## Figures and Tables

**Table 1:** Descriptive statistics of media use in hours per day.

	Min	Max	*M*	*SD*
TV	1	4	1.47	0.74
Smartphone	0	4	0.73	0.88
Tablet	0	3	0.70	0.83
Game console	0	5	1.04	1.14
Computer	0	3	0.29	0.58
Overall media use	1	12	4.22	2.41

Notes. N = 77; Min, minimum; Max, maximum; *M*, mean; *SD*, standard deviation. Overall media use represents the sum of usage times of individual applications per day.

**Table 2:** Pearson correlations between overall media use in hours per day and attentional subdomains.

		(1)	(2)	(3)	(4)	(5)
(1)	Overall media use	-				
(2)	Single demand: selective	0.120	-			
(3)	Single demand: divided	0.017	0.593 **	-		
(4)	Switching demands: selective	0.131	0.740 **	0.761 **	-	
(5)	Switching demands: divided	0.068	0.603 **	0.685 **	0.713 **	-

Notes. N = 61; ** indicate *p*-values significance at an alpha threshold < 0.001.

## Data Availability

The data presented in this study are available on request from the corresponding author. The data are not publicly available due to privacy restrictions.
